# Quercetin inhibits the cAMP/PKA/CREB/glycolysis axis to exert anti-acute lymphoblastic leukemia effects

**DOI:** 10.3389/fphar.2025.1614973

**Published:** 2025-08-04

**Authors:** Qiuling Ma, Wenqing Liu, Yajing Su, Jue Guo, Man Zhang, Jiayuan Guo, Mingxin Liu, Wenbo Dong, Mingwei Li, Bo Wang

**Affiliations:** ^1^ The Second School of Clinical Medicine, Henan University of Chinese Medicine, Zhengzhou, Henan, China; ^2^ Department of Hematology, Henan Province Hospital of Traditional Chinese Medicine (The Second Affiliated Hospital of Henan University of Chinese Medicine), Institute of Hematology, Henan University of Chinese Medicine, Zhengzhou, Henan, China; ^3^ He Nan Province Engineering Research Center of Integrative Medicine for the Prevention and Treatment of Hematological Diseases, Zhengzhou, Henan, China

**Keywords:** acute lymphoblastic leukemia, quercetin, glycolysis, CAMP/PKA/CREB, metabolic reprogramming

## Abstract

Acute lymphoblastic leukemia (ALL), an aggressive hematologic malignancy with high incidence and treatment resistance, demands novel therapeutic approaches. Enhanced glycolysis is a hallmark of metabolic reprogramming in ALL. Quercetin (Que) demonstrates broad antitumor effects by inhibiting glycolysis and cell proliferation. Previous studies have shown that Que significantly suppresses the proliferation of ALL cells and induces apoptosis; however, its mechanistic basis remains elusive. In this study, we demonstrate that Que triggers mitochondrial dysfunction, activating the intrinsic apoptosis pathway and inducing G2/M phase cell cycle arrest. Que markedly reduces glucose uptake, lactate production, and ATP synthesis in ALL cells, suggesting a dual inhibitory effect on both oxidative phosphorylation (OXPHOS) and glycolysis. Mechanistically, Que inhibits the cAMP/PKA/CREB axis, substantially reducing both mRNA and protein levels of glycolytic enzymes (HK2, PFKP, and PKM2). Notably, the PKA-specific agonist Sp-cAMP (50 μmol/L) completely rescues Que-mediated effects. Collectively, Que’s dual pro-apoptotic and anti-proliferative actions through the cAMP/PKA/CREB/glycolysis axis establish a molecular foundation for Que-based ALL therapies.

## 1 Introduction

Acute lymphoblastic leukemia (ALL) is a malignant proliferative disease of lymphocytes with blocked early differentiation, involving the bone marrow, blood, and extramedullary sites. It is primarily classified into B-lineage tumors, T-lineage tumors, and NK cell lineages (Malard and Mohty, 2020). Recent data show an increasing incidence of ALL, with the American Cancer Society estimating 6,550 new cases and 1,330 deaths in 2024 (Siegel et al., 2024). ALL has a poor prognosis, characterized by low survival rates and high relapse rates. Under risk stratification-oriented combination chemotherapy with multiple cytotoxic drugs, the 5-year survival rate of pediatric patients reaches 90%, but approximately 10%–15% of children experience disease relapse (Pennesi et al., 2022). The 5-year overall survival (OS) rate for adult and elderly patients is only 30%–40%, with a relapse rate as high as 40%–50% (Shah et al., 2021). Relapsed ALL patients have a poor response to traditional salvage therapies and an extremely poor prognosis. In the pediatric population, the OS after first relapse is only 50%, while the event-free survival (EFS) rates after second and third relapses are approximately 25% and 15%, respectively (Brivio et al., 2021). For relapsed adult patients, the OS is only 6 months (Pasvolsky et al., 2023). Therefore, developing novel drug therapies for ALL is clinically important.

The growing application of traditional Chinese medicine (TCM) has demonstrated its therapeutic potential in ALL treatment. Herbal medicines and their bioactive compounds exhibit antitumor activity by inhibiting proliferation, inducing apoptosis, suppressing metastasis, reversing chemoresistance, modulating immunity, and regulating the tumor microenvironment (Faubert et al., 2020). Herbal bioactive compounds represent valuable resources for novel drug development. Therefore, investigating ALL pathogenesis and developing targeted therapies are crucial for improving patient outcomes.

Metabolic reprogramming is a hallmark of cancer cells (Wang et al., 2021), with altered glucose metabolism (the “Warburg effect”) being the most common change - where cancer cells preferentially use glycolysis over oxidative phosphorylation despite oxygen availability (Koppenol et al., 2011). Recent studies have advanced our understanding of glycolysis in ALL. R-2-hydroxyglutarate (D-2-HG), a metabolite of mutated isocitrate dehydrogenase, inhibits glycolysis and blocks ALL progression (Qing et al., 2021). USP1 drives T-ALL progression by promoting aerobic glycolysis through PLK1/LDHA axis regulation (Liu et al., 2023). 1,5-anhydroglucitol (1,5-AG) accelerates B-precursor ALL progression by upregulating PDK4 to enhance glycolysis (H. Zhu et al., 2023). Disruption of the miR-652-5p/Tigar axis inhibits glycolysis and suppresses T-ALL cell growth (Liu et al., 2022). MAGI2-AS3 overexpression inhibits growth and glycolysis while promoting apoptosis in ALL cells (Chen X. G. et al., 2022). CDK9 inhibitors suppress c-Myc-mediated glycolysis to induce apoptosis in B-ALL cells (Huang et al., 2021). Thus, glycolysis inhibition represents an effective therapeutic strategy against ALL.

Our preliminary study employed data mining of ALL herbal prescriptions, identified high-frequency herbs, and used network pharmacology to establish component-target relationships, revealing quercetin (Que) as a core active compound. Que is a natural polyphenolic flavonoid (Zalpoor et al., 2022). It occurs abundantly in TCM herbs like Astragalus membranaceus (huangqi), Oldenlandia diffusa (baihuashe shecao), Salvia miltiorrhiza (danshen), and Scutellaria barbata (banzhi lian). Que demonstrates multiple pharmacological properties, particularly anticancer and anti-inflammatory activities. Que effectively inhibits proliferation across cancer types and ranks among the most potent natural anticancer compounds (Khatoon et al., 2022). Que also suppresses the Warburg effect in cancer cells. For example, Que inhibits glycolysis and metastasis in thyroid cancer (TPC-1 cells) by targeting glycolytic pathways (Li et al., 2022). Que downregulates GLUT1 in breast cancer (MCF7 cells), impairing glycolytic capacity and mitochondrial metabolism (Jia et al., 2018). Que suppresses oral squamous cell carcinoma by inhibiting glycolysis and proliferation via the G3BP1/YWHAZ axis (Hu et al., 2023). Que reduces HK2 levels in hepatocellular carcinoma (HCC), suppressing HK2-dependent glycolysis and AKT/mTOR signaling to inhibit progression (Wu et al., 2019). Que alters lactate/pyruvate metabolism in cervical cancer (HeLa cells), exerting cytotoxic and antimetastatic effects (Pani et al., 2021). Furthermore, Que inhibits lymphoma growth by suppressing glycolysis through PI3K-Akt-p53 downregulation (Maurya and Vinayak, 2015).

In summary, ALL is characterized by enhanced glycolytic activity. The natural polyphenolic flavonoid Que regulates key glycolytic enzymes/genes in solid tumors, inhibiting cancer cell proliferation. Our prior studies demonstrated Que’s dose- and time-dependent inhibition of ALL cell proliferation and apoptosis induction. However, the mechanisms underlying Que’s growth-inhibitory effects on ALL cells require further elucidation. Therefore, this study investigates Que’s anti-ALL mechanisms through glycolytic regulation, providing a theoretical basis for Que as a potential therapeutic agent for ALL.

## 2 Materials and methods

### 2.1 Chemicals and reagents

Antibodies against PKA (#5842, 1:1000), p-PKA (#5661, 1:1000), CREB (#9197, 1:1000), p-CREB (#9198, 1:1000), Bcl-2 (#3498, 1:1000), Bax (#5023, 1:1000), Caspase3 (#9662, 1:1000), PARP (#9532, 1:1000), HK2 (#2867, 1:1000), PFKP (#12746, 1:1000), and PKM2 (#4053, 1:1000)were purchased from Cell Signaling Technology (CST) (Beverly, MA, United States). Antibodies against β-actin (66009-1-Ig, 1:20000), and GAPDH (10494-1-AP, 1:5000) were purchased from Proteintech (Wuhan, China). Antibodies against CDK1 (ET1607–51, 1:2000) and cyclin B1 (ET1608–27, 1:5000) were purchased from Huabio (Hangzhou, China). Que (C_15_H_10_O_7_, MW 302.24 g/mol, purity ≥97.97%, batch #BNU332) was purchased from Shanghai Bide Pharmaceutical Technology Co., Ltd. (Shanghai, China). Sp-cAMP (HY-100530C; MCE).

### 2.2 Cell culture

The acute lymphoblastic leukemia cell lines CEM and MOLT-4 were purchased from Wuhan Pulun Sai Life Science Co., Ltd. (product numbers: CL-0328 and CL-0160). Cells were cultured in RPMI-1640 complete medium (Solarbio) supplemented with 10% fetal bovine serum (ShuangRu Biotech). Cells were maintained at 37°C with 5% CO_2_ and 95% humidity in a incubator (Thermo). All cell lines tested negative for *mycoplasma* contamination.

### 2.3 Cell proliferation assays

CEM and MOLT-4 cells were treated in 24-well plates for 48 h with: (a) Que at appropriate concentrations, (b) 50 μM Sp-cAMP (HY-100530C; MCE), (c) or their combination. Then, 100 μL of supernatant was transferred to 96-well plates, followed by addition of 10 μL CCK-8 solution per well. After 3-h incubation, absorbance was measured at 450 nm using a microplate reader (Thermo Scientific). Cell proliferation rate was calculated as:

$$\text{Cell proliferation rate}=\left(\text{Experimental group OD value}-\text{Control group OD value}\right)\;/\text{ Control group OD value}\times 100\%$$

### 2.4 Cell cycle analysis

Cell cycle analysis was performed using a Cell Cycle Staining Kit (Lianke Biotechnology, Hangzhou, China). Cell cycle analysis was performed using flow cytometry and propidium iodide (PI) staining. CEM and MOLT-4 cells were seeded at a density of 1 × 10^5^ cells per well in 6-well plates. After 48 h of treatment with different concentrations of quercetin, the cell suspensions were collected and centrifuged at 1,000 rpm for 5 min. The collected cells were resuspended and fixed in 70% ethanol overnight at 4°C. Subsequently, the cells were washed with cold PBS and centrifuged again at 1,000 rpm for 5 min. The cell pellets were resuspended in a pre-prepared RNase A/PI staining solution (RNase A:PI = 1:9) and incubated at 37°C in the dark for 30 min. The cells were analyzed using flow cytometry (FACS Calibur, BD Biosciences, United States). The experimental results were analyzed using ModFit LT version 5.0 and Prism version 9 for graphing and statistical analysis.

### 2.5 Apoptosis assay

Apoptosis was detected using an Annexin V/PI Apoptosis Kit (Nanjing Yunhe, China). Apoptosis was assessed using Annexin V-FITC/PI staining and flow cytometry. CEM and MOLT-4 cells treated with different concentrations of quercetin and 50 μM Sp-cAMP were analyzed for apoptosis. Briefly, the collected cells were stained using an Annexin-V-FLUOS Staining Kit (Nanjing Yunhe, China), which consisted of 100 μL binding solution, 2 μL Annexin V, and 2 μL PI. The cells were stained in the dark at room temperature for 10 min and then measured using flow cytometry. The experimental results were analyzed using FlowJo version 10.8.1and Prism version 9 for graphing and statistical analysis.

### 2.6 Transmission electron microscopy (TEM)

Select cells in the logarithmic growth phase, adjust the cell count to 1 × 10^5 cells/mL, and inoculate 3 mL into a 25 mL culture flask for cultivation. Wash with PBS, centrifuge at 90 *g* at room temperature for 10 min, and fix the cells with 3% glutaraldehyde overnight at 4°C. Then dehydrate, embed, and fix the cells, and section them. After double staining with 3% uranyl acetate and lead citrate, observe the ultrastructure of the cells under a transmission electron microscope.

### 2.7 RNA preparation and florescent real-time PCR

Total RNA was isolated from cultured cells using an RNA extraction kit (DP431, TIANGEN BIOTECH, Beijing, China), and the purity and concentration of the isolated RNA were assessed using a spectrophotometer (DS-11, Denovix, United States). cDNA was synthesized using the FastKing RT Kit (with gDNase) (KR116, TIANGEN BIOTECH, Beijing, China). RT-PCR was performed in a real-time PCR detection system (CFX96; Bio-Rad) using primers (Sangon Biotech, Shanghai, China) and TransStart^®^ Green qPCR SuperMix UDG reagent kit (AQ111-01, TRANSGEN BIOTECH, Beijing, China). mRNA levels were calculated using the 2^−ΔΔCT^ method, with C-ABL as an endogenous control. The primers were as follows (5′-3′):

C-abl: forward, TGG​AGA​TAA​CAC​TCT​AAG​CAT​AAC​TAA​AGG​T; reverse, GAT​GTA​GTT​GCT​TGG​GAC​CCA.

HK1: forward, CAC​ATG​GAG​TCC​GAG​GTT​TAT​G; reverse, CGT​GAA​TCC​CAC​AGG​TAA​CTT​C.

HK2: forward, TGC​CAC​CAG​ACT​AAA​CTA​GAC​G; reverse, CCC​GTG​CCC​ACA​ATG​AGA​C.

PFKP: forward, GGG​ACG​ATC​ATT​GGC​AGT​G; reverse, GAG​GTA​GGC​GTA​CTT​CTG​CAC.

PKM1: forward, TGC​GAG​CCT​CAA​GTC​ACT​CCA​C; reverse, TCA​CGG​CAC​AGG​AAC​AAC​ACG.

PKM2: forward, GCC​TGC​TGT​GTC​GGA​GAA​G; reverse, CAG​ATG​CCT​TGC​GGA​TGA​ATG.

### 2.8 Western blot assays

After treating CEM and MOLT-4 cells with different concentrations of quercetin for 48 h, the cells were collected. Total protein was extracted using RIPA lysis buffer, and the protein concentration was determined using the BCA assay. Twenty micrograms of total protein were loaded onto an SDS-PAGE gel, separated by electrophoresis, and then transferred to a membrane. The membrane was blocked with 5% skim milk for 2 h, and then incubated with primary antibodies against Bcl-2 (1:1000), Bax (1:1000), Caspase3 (1:1000), PARP (1:1000), CDK1 (1:2000), cyclin B1 (1:5000), PKA (1:1000), p-PKA (1:1000), CREB (1:1000), p-CREB (1:1000), HK2 (1:1000), PFKP (1:1000), PKM2 (1:1000), β-actin (1:20000), and GAPDH (1:5000) overnight at 4°C. After incubation with secondary antibodies for 2 h at room temperature, the blots were visualized using an ECL chemiluminescent detection system.

### 2.9 Mitochondrial membrane potential assay

Collect the cells and add 1 mL of JC-1 staining solution to each well, mix thoroughly, and then incubate at 37°C for 20 min in a cell culture incubator (protected from light). Centrifuge at 1200 r/min for 5 min, discard the supernatant, and wash the cells twice with JC-1 staining buffer (1x). Finally, analyze the samples using a flow cytometer.

### 2.10 Detection of intracellular ATP levels

ATP levels were measured using an ATP assay kit (Beyotime, S0026) following the manufacturer’s instructions. Leukemia cells were collected and lysed with 200 μL lysis buffer for 20 min. The lysate was vortexed to ensure complete lysis and then centrifuged at 12,000 rpm for 5 min at 4°C. The supernatant was collected and kept on ice for further analysis. Protein concentration in the lysate was determined using a BCA protein assay kit. To measure ATP, 100 μL of ATP working solution (1:9) was added to each sample and incubated at room temperature in the dark for 5 min to consume background ATP. Finally, 20 μL of each sample was transferred to wells, and chemiluminescence was measured using a multifunctional microplate reader (Thermo Scientific).

### 2.11 Detection of reactive oxygen species

Intracellular reactive oxygen species (ROS) levels were assessed using a ROS assay kit (Beyotime, S0033S). Leukemia cells were seeded in six-well plates and treated with QUE for 48 h. Cells were then harvested and incubated with 10 μmol/L DCFH-DA (diluted 1:1000 in serum-free medium) at 37°C for 20 min in the dark. After incubation, cells were washed twice with PBS and analyzed by flow cytometry.

### 2.12 Glucose consumption assay

According to the manufacturer’s instructions, we measured glucose consumption using a Glucose Consumption Assay Kit (Beyotime, S0201). CEM and MOLT-4 cells were treated with various concentrations of QUE for 48 h, then collected and resuspended in a lysis buffer. The suspension was transferred to 1.5 mL Eppendorf (EP) tubes, subjected to three freeze-thaw cycles at −20°C to ensure complete lysis, and centrifuged at 12,000 rpm for 30 min at 4°C. The supernatant was collected and kept on ice for analysis. Absorbance was measured at 630 nm using a microplate reader (Thermo Scientific).

### 2.13 Measurement of oxygen consumption rate and extracellular acidification rate

The XF96 Extracellular Flux Analyzer (Agilent Technologies, North Billerica, Massachusetts, United States) is used to detect real-time changes in the extracellular acidification rate (ECAR). Pre-coat the culture plate with Cell-Tak™ and seed CEM and MOLT-4 cells at a density of 3 × 10^4 cells per well and culture overnight, reaching a confluence of 80%–90% after overnight culture. The next day, treat the cells with Que for 48 h. For OCR analysis, sequentially add oligomycin, carbonyl cyanide 4-(trifluoromethoxy) phenylhydrazone (FCCP), and rotenone/antimycin A. For ECAR analysis (glycolytic stress test), sequentially add glucose, oligomycin, and 2-DG (Agilent, Seahorse XF Glycolytic Stress Test Kit, catalog number 103020–100, United States). At the end of the Seahorse experiment, normalize the cell count in each well using Hoechst33342 (Solarbio, Beijing, China).

### 2.14 Bioinformatics analysis

We initiated our study by submitting total RNA samples to Novogene Co., Ltd. (Beijing, China) for transcriptome sequencing and quantitative analysis. Total RNA was extracted using Trizol reagent, and sequencing libraries were prepared following the manufacturer’s protocol using the RNA Library Prep Kit for Illumina. Qualified libraries were pooled and sequenced on an Illumina platform with 150-bp paired-end reads. Raw reads were initially assessed for basic quality metrics using Fastp (0.20.1). Raw reads were processed using BBTools (39.17) and fastp (0.20.1) to trim adapter sequences and filter low-quality reads.

Data analysis was conducted using R (Version 4.4.2). Raw reads were aligned to the human reference genome GRCh38 (hg38) using the Rsubread package (2.18.0), and gene expression was quantified using the featureCounts function. Differential expression analysis was performed using the DESeq2 package (1.46.0). Significance was determined using the adjusted P-value (padj), calculated via multiple hypothesis testing correction to control for false discovery rates. Genes with padj ≤0.05 were considered significantly differentially expressed. Gene expression changes were visualized using the R packages pheatmap (1.0.12) and ggplot2 (3.5.1) to generate heatmaps and volcano plots, respectively. Gene Set Enrichment Analysis (GSEA) was performed using the official GSEA platform (https://www.gsea-msigdb.org/gsea/index.jsp). KEGG pathway enrichment analysis of DEGs was conducted using the Bioinformatics platform (https://www.bioinformatics.com.cn/). Additionally, potential CREB target genes were identified using the GTRD human transcription factor database (http://gtrd20-06.biouml.org/).

### 2.15 Statistical analysis

Statistical analyses were conducted using GraphPad Prism (v10.0; GraphPad Software) and SPSS (v25.0). Normally distributed quantitative data are expressed as mean ± standard deviation (SD). Intergroup differences were analyzed by Student’s t-test or one-way ANOVA. *P* < 0.05 was considered statistically significant.

## 3 Results

### 3.1 The effect of Que on the growth of CEM and MOLT-4 cells

Our previous studies showed that Que inhibits the proliferation of ALL cell lines CEM and MOLT-4, and induces apoptosis in a dose- and time-dependent manner, as confirmed by the CCK-8 assay and Annexin V-FITC/PI double staining.

**FIGURE 1:**
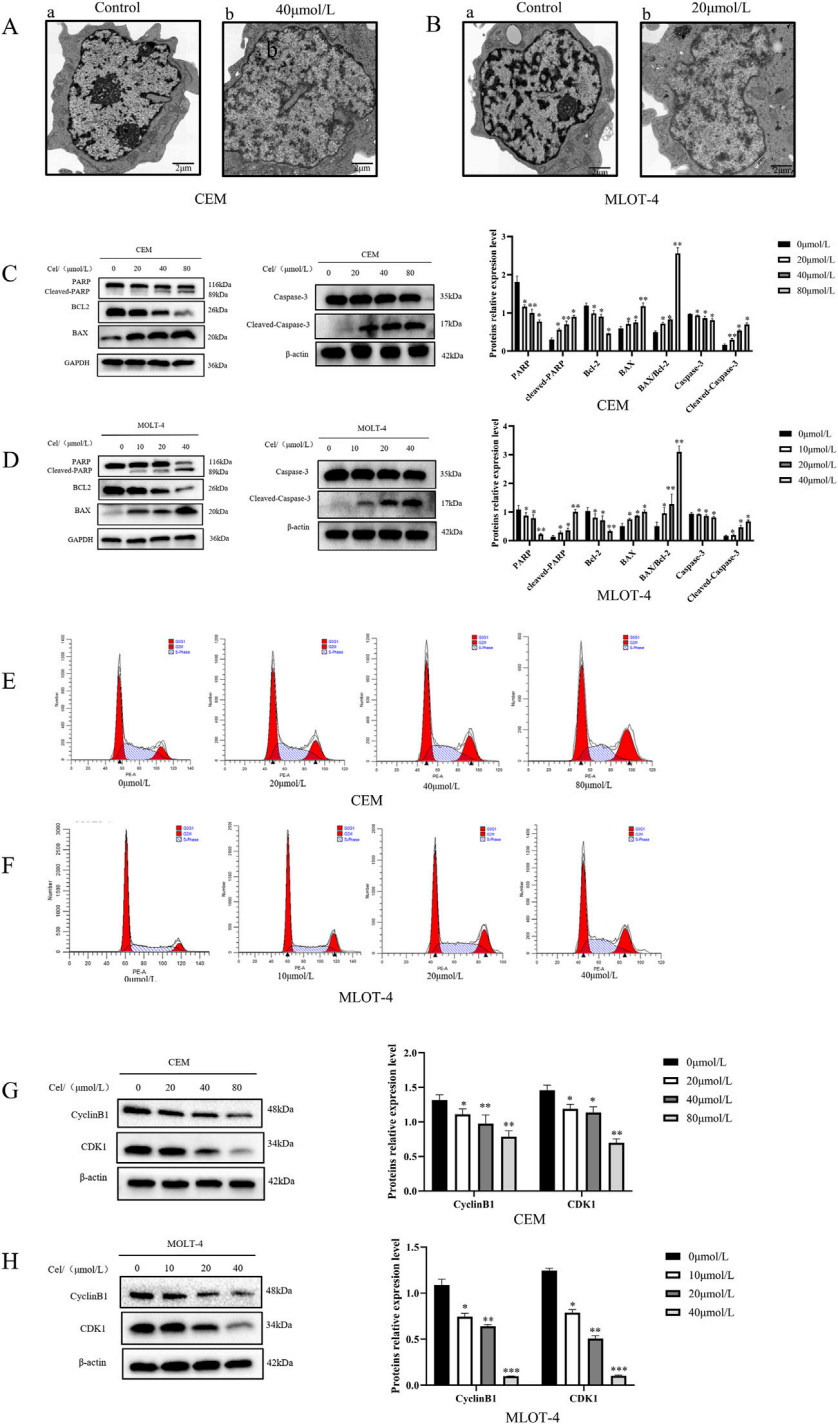
The effect of Que on the growth of CEM and MOLT-4 cells. Cells were treated with Que at specified concentrations. **(A,B)** Transmission electron microscopy was used to observe the submicroscopic cellular structures (×30000 magnification; scale bar = 2 μm). **(C,D)** Western blotting was performed to evaluate the expression of apoptosis-related proteins, with β-actin and GAPDH serving as loading controls. **(E,F)** Cell cycle distribution was analyzed using a NovoCyte flow cytometer and NovoExpress software. **(G,H)** Western blotting was further employed to assess the expression of cell cycle-related proteins with β-actin as the loading control. Data are presented as the mean ± SD of three independent experiments. *p < 0.05,**p < 0.01,**p < 0.001,indicate statistical significance and highly significant differences, respectively.

To further confirm apoptosis, transmission electron microscopy revealed that the control groups of CEM and MOLT-4 cells had normal cell bodies with clear nucleoli, intact nuclear membranes, and evenly distributed chromatin (Figures 1A-a,B-a). In Que-treated groups, chromatin translocated, condensed, and aggregated at the nuclear membrane, with nucleoli absent, exhibiting classic apoptotic features (Figures 1A-b,B-b). Western blotting was then used to analyze the expression of apoptosis-related proteins (Figures 1C,D). Increasing Que concentrations elevated the levels of mitochondrial apoptosis-related proteins cleaved-Caspase 3, cleaved-PARP, and pro-apoptotic protein Bax, while reducing anti-apoptotic protein Bcl-2 and raising the Bax/Bcl-2 ratio. The effects of Que on the cell cycle were also examined (Figures 1E,F). After 48 h of Que treatment, the proportion of CEM and MOLT-4 cells in the G2/M phase increased. Analysis of cell cycle proteins (Figures 1G,H) showed that Que significantly reduced the expression levels of Cyclin B1 and CDK1 in CEM and MOLT-4 cells.

### 3.2 The impact of Que on mitochondrial function in CEM and MOLT-4 cells

**FIGURE 2:**
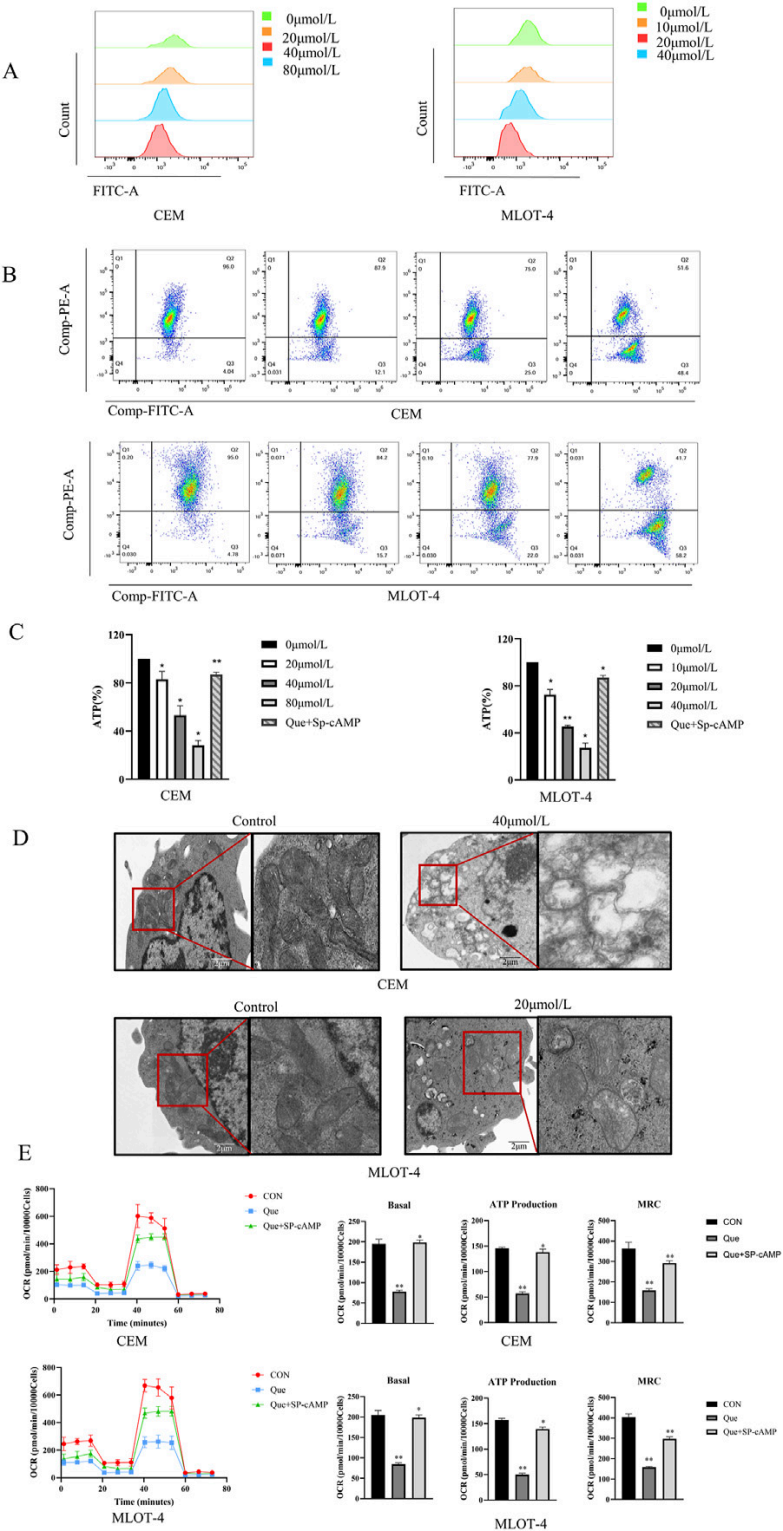
The impact of Que on mitochondrial function in CEM and MOLT-4 cells. Cells were treated with Que at specified concentrations. **(A–C)** The levels of reactive oxygen species (ROS), mitochondrial membrane potential, and intracellular ATP were assessed in CEM and MOLT-4 cells. **(D)** Representative mitochondria in CEM and MOLT-4 cells were observed via TEM (×30,000 magnification; scale bar = 2 μm). **(E)** Real-time changes in mitochondrial oxidative phosphorylation (OCR) were monitored using the XF96 Extracellular Flux Analyzer, and analyses of basal respiration, ATP production, and maximal respiration were performed. Data are presented as the mean ± SD of three independent experiments. *p < 0.05,**p < 0.01 indicate statistical significance and highly significant differences, respectively.

We investigated the effects of Que on mitochondrial function in CEM and MOLT-4 cells by measuring reactive oxygen species (ROS), mitochondrial membrane potential (MMP), and intracellular adenosine triphosphate (ATP) levels. ROS measurements showed that ROS levels in CEM and MOLT-4 cells increased progressively with higher Que concentrations (Figure 2A). After 48 h of Que treatment, the MMP of CEM and MOLT-4 cells was significantly reduced, indicating early apoptosis (Figure 2B). Que treatment also reduced ATP levels in CEM and MOLT-4 cells in a concentration-dependent manner (Figure 2C). Electron microscopy revealed that mitochondria in the control group had normal morphology and number, appearing oval or rod-shaped with neatly arranged cristae. In the Que group, mitochondria were fewer, swollen, vacuolated, and had ruptured membranes, indicative of apoptosis (Figure 2D).

We also assessed the impact of Que on mitochondrial oxidative phosphorylation in CEM and MOLT-4 cells. After 48 h of Que treatment, changes in the oxygen consumption rate (OCR) were monitored using the Seahorse Extracellular Flux Analyzer. After adding oligomycin (an ATP synthase inhibitor), FCCP (which uncouples oxygen consumption from ATP production and maximizes OCR), and rotenone/antimycin A (which inhibit the electron transport chain), the data revealed that Que reduced basal OCR, decreased ATP production in the presence of oligomycin, and suppressed maximum respiratory capacity (MRC) under FCCP stimulation (Figure 2E). These results demonstrate that Que inhibits ATP production and mitochondrial respiratory capacity in CEM and MOLT-4 cells.

### 3.3 The impact of Que on glycolysis in CEM and MOLT-4 cells

**FIGURE 3:**
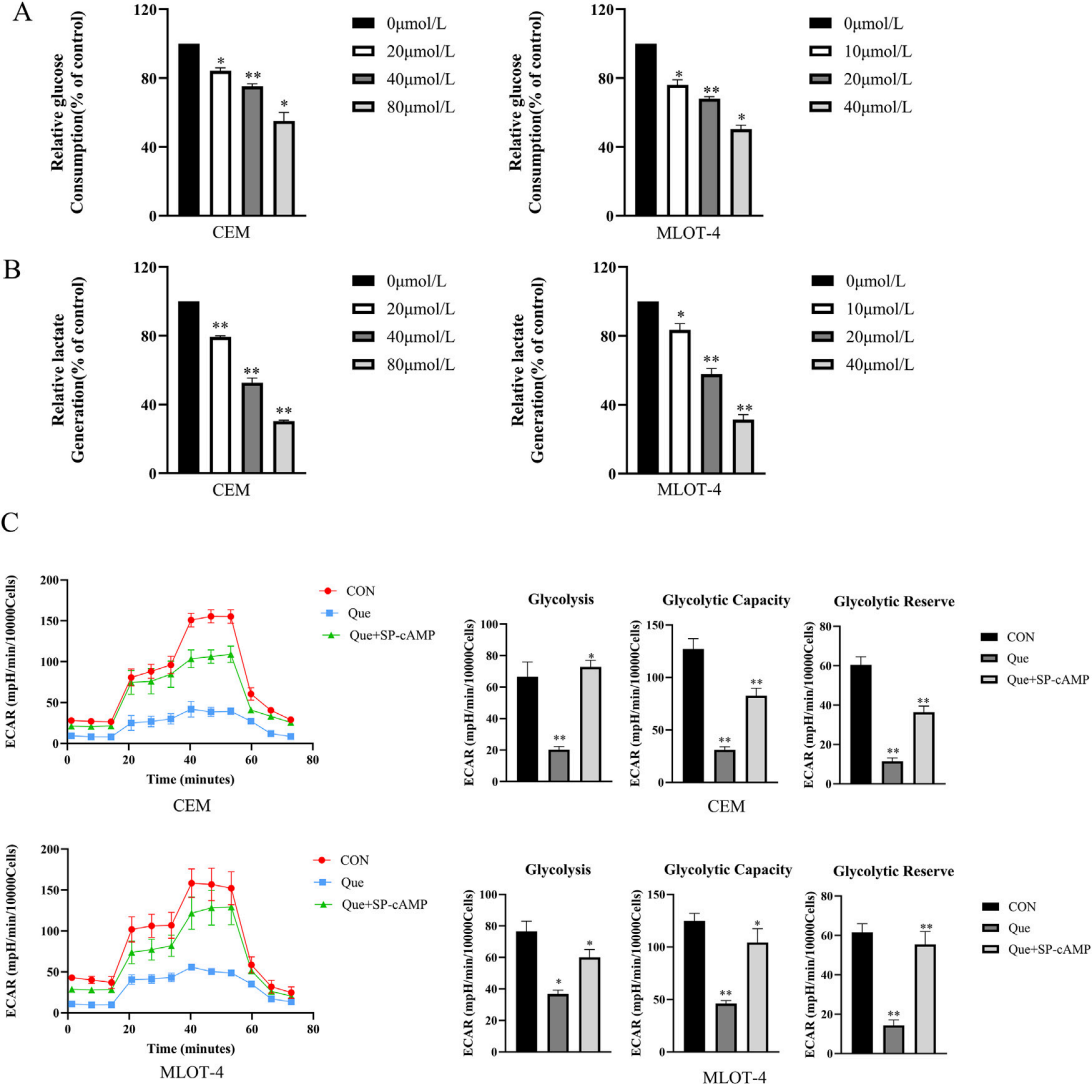
Que inhibits glycolysis in CEM and MOLT-4 cells. Cells were treated with Que at specified concentrations. **(A)** Glucose consumption in CEM and MOLT-4 cells was measured. **(B)** Lactate production in CEM and MOLT-4 cells was detected. **(C)** Real-time changes in extracellular acidification rate (ECAR) were monitored using the XF96 Extracellular Flux Analyzer, and analyses of glycolysis, glycolytic capacity, and glycolytic reserve were performed. Data are presented as the mean ± SD of three independent experiments. *p < 0.05,**p < 0.01 indicate statistical significance and highly significant differences, respectively.

We subsequently examined the effects of Que on glycolysis in CEM and MOLT-4 cells. Glucose uptake and lactate production are key indicators of cellular glycolytic activity. Thus, we evaluated the effects of Que on glucose uptake and lactate production. The results showed that Que treatment significantly reduced glucose uptake and lactate production in a dose-dependent manner in CEM and MOLT-4 cells (Figures 3A,B). To evaluate the effects of Que on the extracellular acidification rate (ECAR), cells were pre-treated with Que for 48 h, glucose-starved, then exposed to glucose, followed by oligomycin to stimulate maximum glycolytic activity. The assay was finalized by adding 2-deoxy-D-glucose (2-DG) to inhibit glycolysis. The data showed that Que inhibited ECAR and, under oligomycin treatment, suppressed glycolytic capacity and reserve in response to oligomycin and FCCP (Figure 3C). These findings suggest that Que suppresses glycolytic activity in CEM and MOLT-4 cells.

### 3.4 The impact of Que on glycolytic key rate-limiting enzymes in CEM and MOLT-4 cells

**FIGURE 4:**
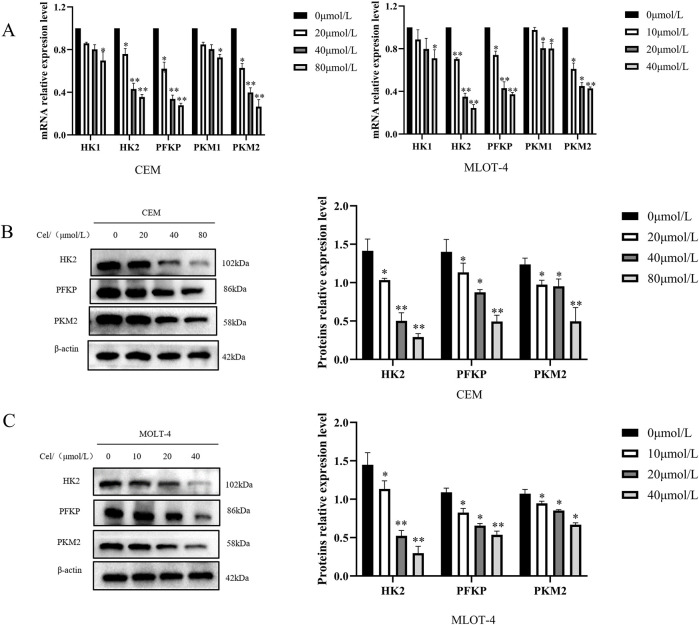
Que inhibits key glycolytic rate-limiting enzymes in CEM and MOLT-4 cells. Cells were treated with Que at specified concentrations. **(A)** The activity of key rate-limiting enzymes involved in glucose metabolism was assessed by Real Time Quantitative (RT-qPCR). **(B,C)** The protein levels of glycolysis-related proteins were analyzed by Western blotting, with β-actin serving as a loading control. Data are presented as the mean ± SD of three independent experiments. *p < 0.05,**p < 0.01 indicate statistical significance and highly significant differences, respectively.

To investigate how Que inhibits mitochondrial respiration and glycolysis, we evaluated the activity of key rate-limiting enzymes in glucose metabolism, including Hexokinase (HK- 1/2), Phosphofructokinase P (PFKP), and Pyruvate Kinase M1/M2 (PKM1/2), in Que-treated CEM and MOLT-4 cells. The results showed that Que treatment decreased the activity of HK-2, PFKP, and PKM2 in both cell lines in a dose-dependent manner, while having no significant impact on HK-1 and PKM1 activity (Figure 4A). We also measured the protein levels of these enzymes and found that Que reduced HK-2, PFKP, and PKM2 expression in a dose-dependent manner in both CEM and MOLT-4 cells (Figures 4B,C). These findings indicate that Que inhibits mitochondrial respiration and glycolysis by suppressing the activity and expression of HK-2, PFKP, and PKM2 in CEM and MOLT-4 cells.

### 3.5 Que exerts its anti- ALL effects through the cAMP/PKA/CREB signaling pathway

**FIGURE 5:**
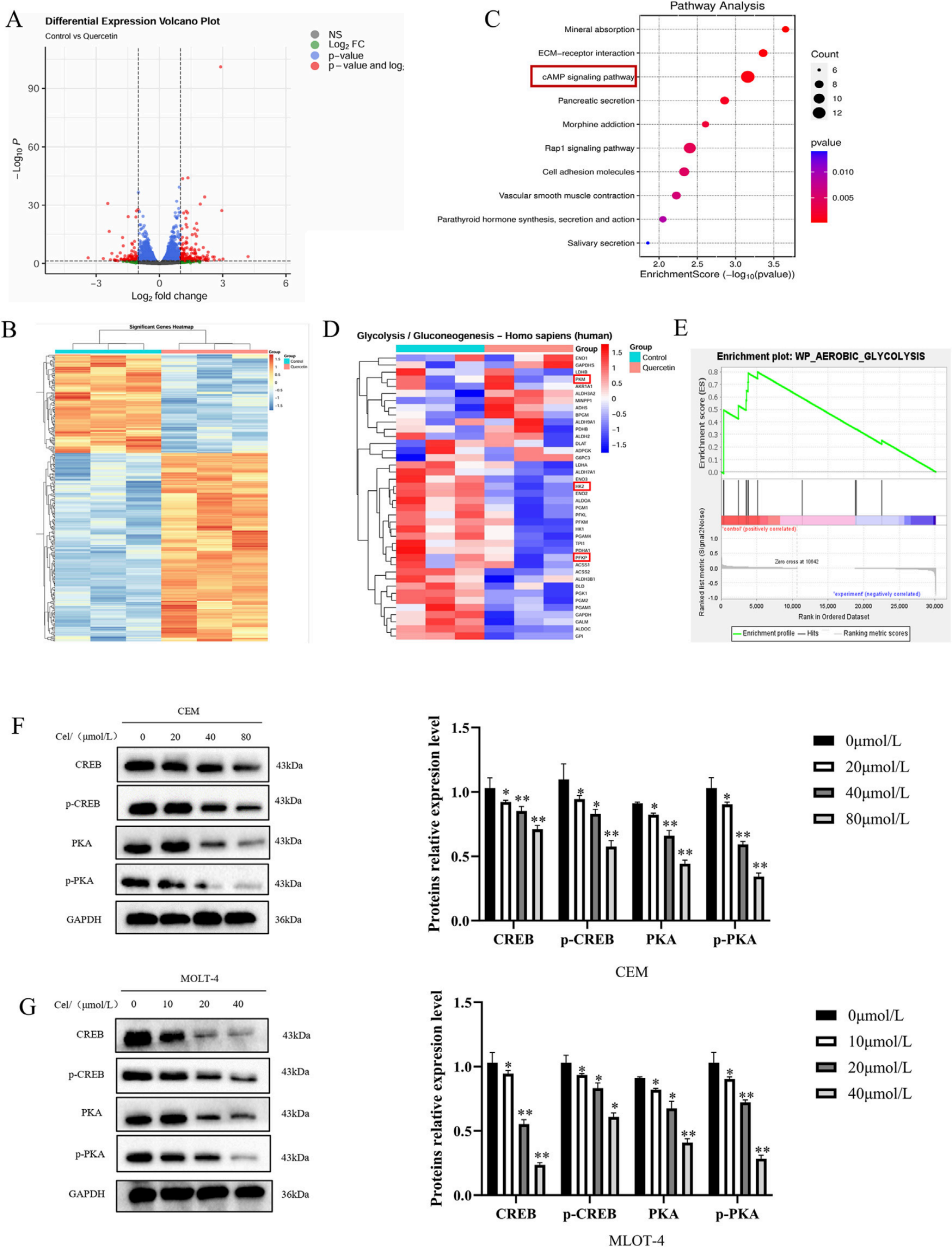
Que exerts its anti-ALL effects through the cAMP/PKA/CREB signaling pathway. Cells were treated with Que at specified concentrations. **(A–E)** Transcriptomic sequencing analysis was performed. **(A)** Volcano plot: Differentially expressed genes were plotted as a volcano plot, with each point representing a gene. **(B)** Heatmap: Differentially expressed genes were visualized using a heatmap. **(C)** KEGG enrichment analysis: The top ten enriched pathways are shown. **(D)** Heatmap: Differentially expressed genes enriched in the glycolysis pathway were plotted as a heatmap. **(E)** GSEA analysis revealed enrichment in the glycolysis pathway. **(F,G)** Western blotting was used to analyze the protein levels of components in the cAMP/PKA/CREB signaling pathway, with GAPDH serving as a loading control. Data are presented as the mean ± SD of three independent experiments. *p < 0.05,**p < 0.01 indicate statistical significance and highly significant differences, respectively.

Based on the antitumor activity and effects on glycolysis of Que on ALL cells, we aimed to explore the underlying molecular mechanisms. Consequently, we performed RNA-seq on MOLT-4 cells treated with Que (20 μmol/L) for 48 h, identified differentially expressed genes, and constructed a volcano plot (Figure 5A), where 182 genes were significantly upregulated, and 95 genes were downregulated (|log2(FC)| > 1 & padj <0.05). A heatmap of these genes was also generated (Figure 5B). KEGG pathway enrichment analysis was then performed on the differentially expressed genes (Figure 5C). KEGG analysis indicated that Que primarily influences pathways such as Mineral absorption, ECM-receptor interaction, and the cAMP signaling pathway. The cAMP (cyclic adenosine monophosphate) signaling pathway is a key intracellular signaling mechanism, with the cAMP/PKA/CREB pathway being especially significant. cAMP, functioning as a second messenger, activates protein kinase A (PKA). Activated PKA phosphorylates CREB (cAMP response element-binding protein), thereby regulating the transcription of various target genes. Using the GTRD human transcription factor database, we identified CREB as a common transcription factor for the HK-2, PFKP, and PKM2 genes. GSEA analysis revealed that differentially expressed genes could be enriched in the glycolysis pathway. A heatmap of these genes is shown (Figures 5D,E). The left side represents the control group, and the right side represents the Que-treated group. Red indicates upregulated gene expression, and blue indicates downregulated gene expression, with the intensity of the color reflecting the degree of upregulation or downregulation. We hypothesize that Que suppresses glycolysis and exerts anti-ALL effects by inhibiting the cAMP/PKA/CREB signaling pathway, thereby modulating the transcription of HK-2, PFKP, and PKM2. To validate this hypothesis, we performed *in vitro* experiments. Results showed that Que treatment caused a dose-dependent reduction in PKA, p-PKA, and p-CREB levels in CEM and MOLT-4 cells (Figures 5F,G), indicating that Que inhibits PKA activity and disrupts CREB phosphorylation.

**FIGURE 6:**
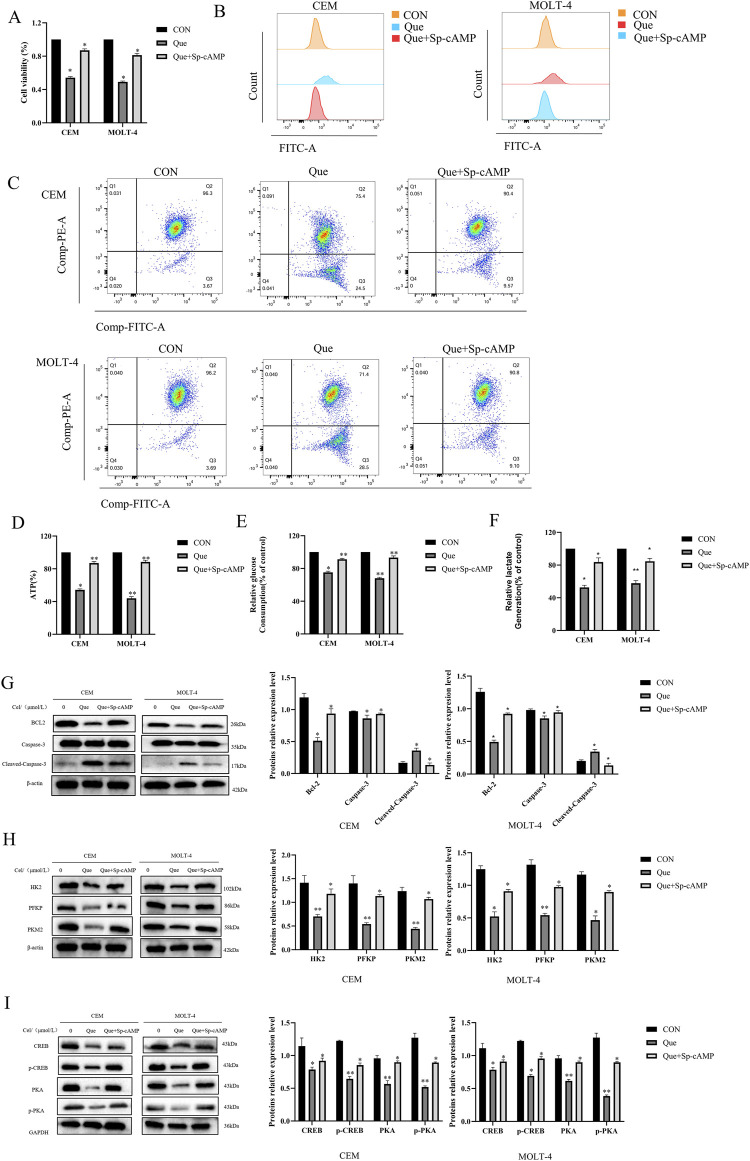
Sp-cAMP attenuates the glycolysis-inhibitory effects of Que in CEM and MOLT-4 cells. After treating CEM and MOLT-4 cells with specified concentrations of Que and Sp-cAMP, **(A)** the impact on cell proliferation was determined using the CCK-8 assay. **(B–F)** The effects on intracellular ROS levels, mitochondrial membrane potential, ATP content, glucose uptake, and lactate production were evaluated. **(G-I)** Western blot analysis was performed to examine the expression of apoptosis-related proteins, glycolysis-related proteins, and proteins in the cAMP/PKA/CREB pathway, with β-actin and GAPDH as loading controls. Data are presented as the mean ± SD of three independent experiments. *p < 0.05,**p < 0.01 indicate statistical significance and highly significant differences, respectively.

Sp-cAMP, a potent and metabolically stable PKA activator, resists degradation by phosphodiesterases (PDEs), thereby sustaining intracellular cAMP levels and prolonging PKA activation. Combined treatment with 50 μmol/L Sp-cAMP and Que restored the expression levels of PKA, p-PKA, p-CREB, HK-2, PFKP, and PKM2 (Figures 6G–I). Furthermore, Sp-cAMP reversed quercetin’s anti-proliferative effects on ALL cells (Figure 6A). Notably, Sp-cAMP counteracted quercetin-induced glycolysis inhibition in ALL cells, restoring glycolytic activity to baseline levels (Figures 6B–F). These findings suggest that quercetin’s inhibitory effect on glycolysis is primarily mediated through the cAMP/PKA/CREB pathway.

## 4 Discussion

Que is a bioflavonoid compound extracted from traditional Chinese medicinal herbs such as fresh small thistle, half-branch lotus, and oldenlandia diffusa, and is one of the most abundant polyphenolic flavonoids. Studies demonstrate that Que has strong anti-cancer effects against various cancer cells, including leukemia cells, making it one of the most potent known anti-cancer agents (Xiao et al., 2022). Our previous research, using CCK-8 and Annexin V-FITC/PI staining, confirmed that Que inhibits the growth of ALL cell lines CEM and MOLT-4 in a time- and dose-dependent manner and induces apoptosis. Transmission electron microscopy further revealed classic apoptotic features in the Que-treated group, confirming cellular apoptosis. Apoptosis primarily occurs via two pathways: the extrinsic apoptotic pathway and the intrinsic (mitochondrial) pathway (Newton et al., 2024). Our results show that Que-induced apoptosis is associated with increased reactive oxygen species (ROS) production and reduced mitochondrial membrane potential. Electron microscopy also revealed alterations in mitochondrial number and morphology. Our study revealed that Que downregulated Bcl-2 expression and upregulated Bax, p-Caspase-3, and p-PARP expression, leading to a significant increase in the Bax/Bcl-2 ratio, consistent with activation of the mitochondrial apoptotic pathway. These findings suggest that Que induces apoptosis in CEM and MOLT-4 cells via the mitochondrial pathway. Regulation of the cell cycle and induction of apoptosis are both critical for leukemia treatment, with apoptosis often associated with cell cycle arrest (Wang et al., 2015). Our findings confirm that Que induces G2/M phase cell cycle arrest and downregulates the expression of G2/M phase-related proteins Cyclin B1 and CDK1.

One key characteristic of cancer cells is metabolic reprogramming, with glucose metabolism abnormalities being among the earliest and most common. This phenomenon, known as the “Warburg effect,” involves malignant tumor cells shifting their primary ATP production from oxidative phosphorylation (OXPHOS) to aerobic glycolysis, even in the presence of oxygen. For the first time, we have confirmed the effect of Que on mitochondrial function and glycolysis in ALL cells. Que reduced mitochondrial membrane potential, glucose uptake, lactate and ATP production, and increased ROS production in ALL cells. Using the Seahorse XFe96 extracellular flux analyzer, we found that Que treatment downregulated both the ECAR and OCR, indicating that Que inhibits glycolysis and induces mitochondrial dysfunction.

We propose that Que might disrupt aerobic glycolysis by altering the expression of metabolism-related genes. Glycolysis is primarily controlled by key rate-limiting enzymes, such as hexokinase (HK), phosphofructokinase (PFK), and pyruvate kinase (PK) (Patra et al., 2013). Notably, HK-2, PFKP, and PKM2 are closely linked to cancer cells, exhibiting significant upregulation in tumors (Xu and Herschman, 2019). Since Que inhibits both glycolysis and OXPHOS, we propose that it may affect the activity of HK2, PFKP, and PKM2. Our findings reveal that Que reduces both the mRNA and protein expression of these enzymes in ALL cells. Studies have shown that these key rate-limiting enzymes not only catalyze glycolysis but also affect mitochondrial function, regulate apoptosis, and modulate the cell cycle. HK-2 and PFKP bind to the voltage-dependent anion channel (VDAC) on the outer mitochondrial membrane. Mitochondrial VDAC serves as a channel for binding and release of apoptotic molecules, such as cytochrome c and BCL2-associated protein (Bax) (Shoshan-Barmatz et al., 2010). The binding of HK-2 and PFKP to VDAC activates their phosphorylation, promoting ATP production by mitochondria, while also preventing pro-apoptotic molecules (e.g., Bax) from binding to VDAC and initiating mitochondrial apoptosis (Xu et al., 2017; Zhou et al., 2018). PKM2 exists primarily as high-activity tetramers and low-activity dimers. The tetrameric form of PKM2 regulates glycolysis, while the low-activity dimer, which loses its catalytic function, translocates to the nucleus to exert protein kinase activity, influencing transcription factors and signaling pathways that promote tumor development (Zhu et al., 2021). PKM2 upregulates gene expression by binding to transcription factors such as HIF-1α, β-catenin, and STAT3, promoting cell growth and regulating the cell cycle. PKM2 interacts with the mitochondrial fusion protein MFN2 to promote mitochondrial fusion, protect mitochondrial function, and enhance OXPHOS (Feng et al., 2013; Li et al., 2019). Therefore, the metabolic shift from glycolysis to OXPHOS induced by the absence of HK-2, PFKP, and PKM2 is accompanied by changes in mitochondrial morphology, increased ROS production, and the release of pro-apoptotic molecules. These changes are signs of mitochondrial dysfunction and apoptosis, consistent with our experimental results (Chen J. et al., 2022; Thomas et al., 2022; Tretter et al., 2016; Zhou et al., 2019). Since these glycolytic enzymes also have non-glycolytic functions, Que, a potential multi-target glycolytic inhibitor, shows promise as an effective anticancer drug.

cAMP, the first identified intracellular second messenger, plays a crucial role in cellular signal transduction (Jiang et al., 2021). cAMP regulates the transcription of target genes through protein kinase A (PKA) and its downstream effectors, including cAMP response element-binding protein (CREB). This signaling cascade modulates gene expression in response to extracellular signals, influencing various cellular functions and adaptations (Zambon et al., 2005). This study reports for the first time that Que plays a pivotal role in acute lymphoblastic leukemia (ALL) by targeting the cAMP/PKA/CREB/glycolysis axis. Specifically, we found that Que reduces the protein levels of PKA and phosphorylated PKA, inhibits CREB phosphorylation, and suppresses the expression of glycolytic enzymes HK-2, PFKP, and PKM2. Notably, various experimental data further validate our findings, showing that the PKA activator Sp-cAMP effectively counteracts Que. The cAMP–PKA–CREB pathway regulates mitochondrial function and promotes apoptosis in lymphoma cells through a mitochondria-dependent pathway, decreasing Bcl-2 expression and increasing Bax expression (Mamani-Matsuda et al., 2004; Zhang et al., 2020; Zhang et al., 2008). These observations align with our experimental data.

**FIGURE 7:**
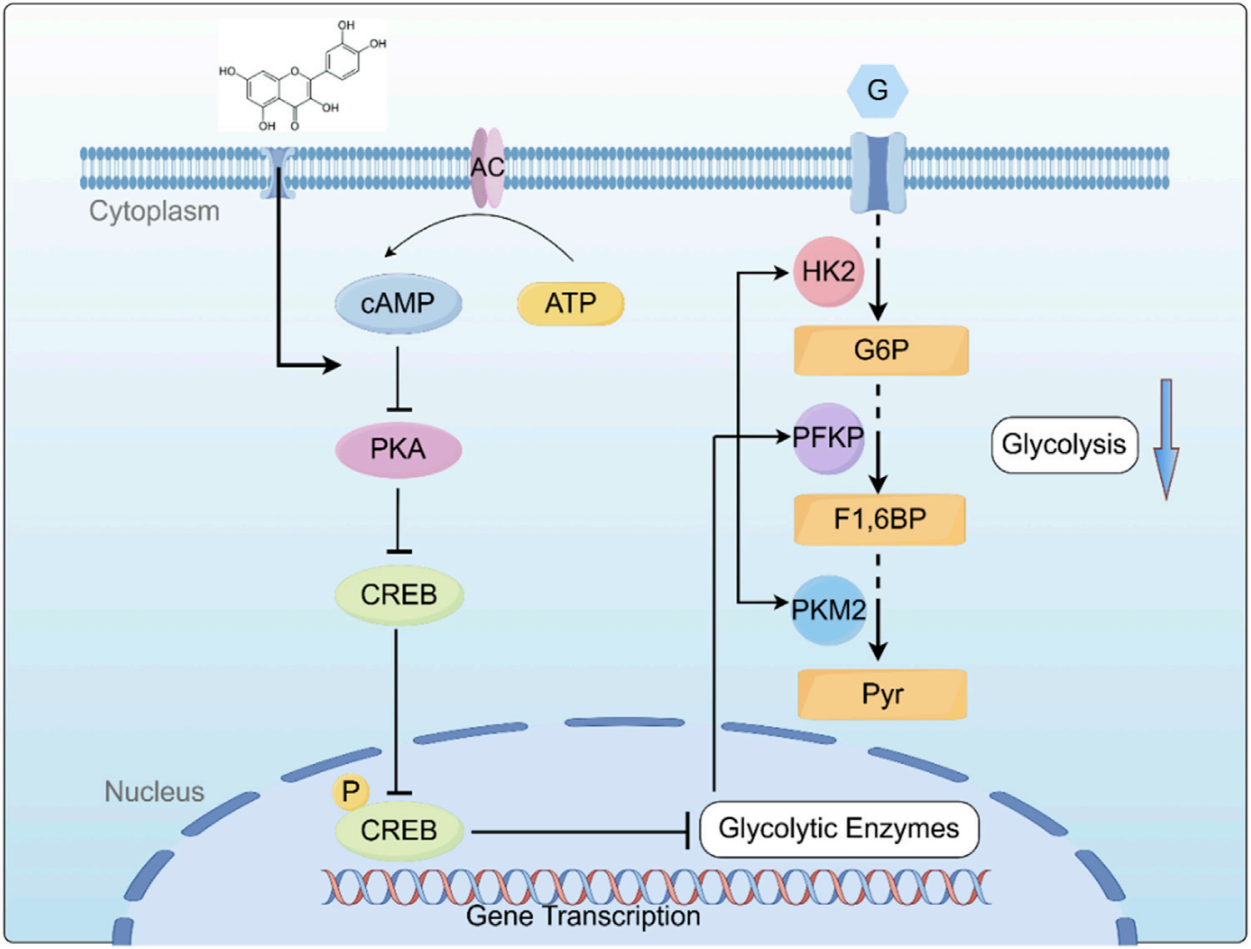
Graphical summary of the mechanism by which QR inhibits ALL Cell growth by inhibiting the cAMP/PKA/CREB/Glycolysis Axis. cAMP: cyclic AMP; PKA: protein kinase A; CREB: cAMP-response element-binding protein; HK2: Hexokinase II; PFKP: Phosphofructokinase P; PKM2: Pyruvate Kinase M2; G: Glucose; G6P: Glucose-6-phosphate; F1,6BP: Fructose-1,6-Bishosphate; Pyr: P.yruvate. Created using Figdraw (www.figdraw.com).

Based on our key findings, we constructed a schematic diagram (Figure 7) delineating the molecular mechanism underlying Que induced cell death in ALL. This study demonstrates that Que specifically targets the cAMP/PKA/CREB signaling axis, dually modulating both glycolytic metabolic reprogramming (via HK-2/PFKP/PKM2 downregulation) and mitochondrial dysfunction, thereby synergistically suppressing cell proliferation, triggering apoptosis, and inducing G2/M phase arrest. This study provides the first systematic elucidation of quercetin’s multi-target anti-leukemia mechanisms through metabolic reprogramming, offering novel perspectives for developing nature-derived targeted therapies against ALL. Despite these findings, certain limitations remain: all data were derived from *in vitro* experiments. Future studies will employ patient-derived xenograft (PDX) models to validate Que’s *in vivo* efficacy and utilize primary ALL cells to assess clinical relevance, aiming to better support ALL clinical treatment.

## Data Availability

The original contributions presented in the study are included in the article/supplementary material, further inquiries can be directed to the corresponding authors.
